# Cannabis use among adolescents and young adults in Germany: Study results and prevention measures offered by the Federal Institute of Public Health

**DOI:** 10.25646/13458

**Published:** 2025-09-24

**Authors:** Stephanie Eckhardt, Anika Nitzsche, Boris Orth

**Affiliations:** 1 Federal Institute of Public Health, Unit T 4 – Addiction Prevention, Cologne, Germany; 2 Federal Institute of Public Health, Unit Q 3 – Evaluation, Methods, Research Data, Cologne, Germany

**Keywords:** Cannabis use, Germany, Temporal trends, Prevention, Adolescents, Young adults

## Abstract

**Background:**

With the passage of the Consumer Cannabis Act, cannabis was partially legalised on 1 April 2024 in Germany. Cannabis remains prohibited for adolescents under the age of 18. This article analyses how the prevalence of cannabis use among young people in Germany has developed up to this point and presents prevention measures and funding projects offered by the Federal Institute of Public Health (BIÖG, formerly the Federal Centre for Health Education, BZgA).

**Methods:**

Based on representative studies by the BIÖG, sociodemographic differences in 2023 and trends in the 12-month prevalence of cannabis use between 2008 and 2023 are analysed for 12- to 17-year-old adolescents and 18- to 25-year-old young adults. Current prevention measures are systematically presented.

**Results:**

The 12-month prevalence of cannabis use remained relatively stable among female and male adolescents before partial legalisation, but increased significantly among young women (2008: 8.3 %; 2023: 19.4 %) and men (2008: 14.8 %; 2023: 26.9 %). Cannabis prevention measures focus on the school setting and digital services.

**Conclusions:**

The effects of partial legalisation on cannabis use among adolescents and young adults must be evaluated in future studies. The BIÖG offers well-founded information on cannabis, its effects and health risks, as well as digital counselling services and self-tests for different target groups, and is continuously expanding its services.

## 1. Introduction

With the entry into force of the Consumer Cannabis Act (KCanG^1^) on 1 April 2024, the personal cultivation of cannabis is legal for adults in Germany, subject to the conditions laid down by law. In addition, the communal, non-commercial personal cultivation of cannabis in cultivation associations has been permitted under certain conditions since 1 July 2024. These cultivation associations may distribute cannabis to adults for personal consumption in a controlled manner. However, cannabis remains prohibited for young people under the age of 18. In addition to curbing cannabis-related crime, the law aims to strengthen cannabis-related education and prevention as well as the protection of children and young people. The law therefore also stipulates the expansion of the education and prevention work of the Federal Institute of Public Health (BIÖG; still referred to in the text of the law as the Federal Centre for Health Education^2^) and the evaluation of the aforementioned objectives.

It is well known that the first use of cannabis often takes place in adolescence [1]. Like other behaviours that are harmful to health, substance use often begins in adolescence, as it is used as a means of coping with certain steps on the path to adulthood, such as separating from parents and gaining autonomy, developing one’s own identity (including gender identity) or forming friendships and intimate relationships [2, 3]. Young people are not only at risk of developing (problematic) substance use during adolescence, they are also particularly vulnerable to the adverse effects of substance use due to their ongoing growth and maturation phase [4, 5]. Studies also show a dose-dependent link between cannabis use and mental disorders such as depressive disorders, suicidality, bipolar disorders, anxiety disorders and the harmful use of other substances [6]. In addition, the onset of psychosis can be promoted, especially when consuming highly potent cannabis strains [7]. Over time, lung function may be reduced and psychosocial development may be impaired [8, 9]. In a review, the authors conclude that cannabis has potentially harmful effects on cognitive abilities, the brain and educational outcomes (e.g. intelligence quotient, verbal learning, higher educational qualifications), but that cognitive impairments appear to improve again with sustained abstinence [10]. In addition to these and other health risks, repeated use can also lead to addiction [11]. It is estimated that around 9 % of all cannabis users develop an addiction [12]. If cannabis use began in adolescence, the likelihood of addiction is higher, especially if it was used daily [13, 14]. A representative study of young people aged between 12 and 18 in Germany shows a prevalence of cannabis dependence (according to the criteria of the Diagnostic and Statistical Manual of Mental Disorders, Fourth Edition, DSM-IV [15]) of 11.7 % among adolescents who had used cannabis in the last twelve months [16].

An important public health objective is therefore to promote abstinence, especially among young people, to delay the onset of consumption as far as possible into the second decade of life, and to keep prevalence among adults as low as possible.

The BIÖG contributes to this by focusing on early education and raising awareness among young people about the risks of substance use, as well as strengthening resilience and responsible use of substances. In accordance with the principle of subsidiarity, the BIÖG has the task of informing professionals about measures taken by the Federal States and referring those seeking advice to municipal counselling centres. Important prerequisites for this include the provision of training courses for educational professionals and strong networking with local services.

**Key messages**

► In 2023, one in fifteen adolescents aged between 12 and 17 (6.7 %) and one in four young adults aged between 18 and 25 (23.5 %) had used cannabis in the previous twelve months.
► In the fifteen years prior to the partial legalisation of cannabis (2008 – 2023), cannabis use among adolescents changed only slightly.
► Among young women and young men, there was a significant increase in cannabis use in the 15 years prior to partial legalisation (2008 – 2023).
► The BIÖG promotes prevention measures implemented by multipliers. The focus is on universal prevention (i.e. prevention for all) in the school setting.
► The BIÖG’s digital services range from general information and education to individual counselling for young people, adults and multipliers.

In order to develop appropriate prevention measures, it is necessary to know how cannabis use among young people in Germany currently stands and how it is changing in the long term. To this end, the BIÖG collects its own data. In this article, this data is used to first analyse sociodemographic differences in cannabis use among adolescents and young adults aged 12 to 25 in 2023. It then presents trend analyses of cannabis use among adolescents and young adults for the period from 2008 to 2023. The data currently available describe the development and situation prior to the entry into force of the new Consumer Cannabis Act. Finally, the article provides an overview of the BIÖG’s current prevention measures relating to cannabis use among young people, classifies them in terms of their access routes or target group specificity, and provides information on the status of quality assurance and evaluation.

## 2. Methods

### 2.1 Data basis and data weighting

The data is based on representative surveys conducted by the BZgA (now BIÖG) from 2008 to 2023 on substance use among adolescents and young adults in Germany. Specifically, these were the Drug Affinity Studies from 2008, 2011, 2015, 2019 and 2023, as well as the Alcohol Surveys conducted in 2010, 2012, 2014, 2016, 2018 and 2021, in which identical core questions on cannabis use were asked. Adolescents and young adults aged 12 to 25 were surveyed using computer-assisted telephone interviews (CATI). Up to and including 2012, the surveys were conducted exclusively via landline telephones. In 2014, an additional survey via mobile telephone was tested for the first time. The trend analyses in this article are based entirely on surveys conducted using the dual-frame approach from 2015 onwards. This means that two random samples were drawn independently of each other from the two selection frames of the Working Group of German Market and Social Research Institutes (Arbeitskreis Deutscher Markt- und Sozialforschungsinstitute, ADM) for landline and mobile phone numbers and evaluated together [17]. The mix ratio of landline and mobile phone samples was set at 70 % to 30 % from 2015 to 2019 and at 60 % to 40 % from 2021 onwards.

In order to compensate for different selection probabilities due to the sample design, the weighting took into account the number of landline and mobile phone numbers at which the respondents could be reached, as well as the number of 12- to 25-year-olds in the household. In the case of dual-frame samples, a ratio of 1 to 1.5 was used for the number of all landline and mobile phone numbers in the two selection frames (design weighting). The sample was then adjusted to the age, gender and regional distribution of 12- to 25-year-olds in the Federal Republic of Germany (according to the population update by the German Federal Statistical Office and the microcensus) and, from 2015 onwards, to their educational distribution.

The response rates fell from 68.4 % (2008) to 38.6 % (2023) for landline samples and from 32.0 % (2015) to 29.7 % (2023) for mobile phone samples.

The data collections for 2010, 2012 and 2014 were carried out by Kantar-Health GmbH, while all other surveys were carried out by forsa, Gesellschaft für Sozialforschung und statistische Analysen mbH.

Further methodological details on the individual studies can be found in the respective research studies.

### 2.2 Survey instruments

#### Cannabis use (12-month prevalence)

The adolescents and young adults who stated in the Drug Affinity Studies or Alcohol Surveys that they had tried marijuana or hashish or cannabis at least once were asked how often they had used marijuana or hashish or cannabis in the last twelve months. The five response categories available were ‘not at all’, ‘once’, ‘twice’, ‘three to ten times’ and ‘more often’. The 12-month prevalence of cannabis use corresponds to the percentage of respondents who had used cannabis at least once in the last twelve months prior to the interview, i.e. their last use of this substance was no longer than twelve months ago.

#### Sociodemographic characteristics

Gender, age, education and migration background were used as sociodemographic characteristics. Gender was recorded with the question ‘Are you male or female?’. From 2021 onwards, interviewers were also able to select the category ‘diverse’ if respondents so wished. Education was grouped into two categories (high vs. medium/low) for the purposes of the analysis, with young people attending grammar school forming the ‘high’ category and young people attending primary, comprehensive, secondary and other schools or in vocational training forming the other category. Among young adults, respondents with a university entrance qualification were assigned to the highest education category and all others to the medium/low category. Migration background was recorded by asking in which country the respondent themselves and their mother and father were born. In addition, the nationality of the respondents themselves and their parents was also asked. The information was used to form a dichotomous variable in such a way that a migration background was assumed if the respondent or at least one of their parents was born in a country other than Germany or had a nationality other than German.

### 2.3 Statistical analyses

Data from the 2023 Drug Affinity Study was used to analyse sociodemographic differences in cannabis use. For 12- to 17-year-olds and 18- to 25-year-olds, the estimated 12-month prevalence of cannabis use and the corresponding 95 % confidence intervals were determined for the total population and within gender groups. Within these groups, further estimates and 95 % confidence intervals were calculated for the various characteristics of age, education and migration background. Due to the low number of cases, results for people with the gender ‘diverse’ were not reported separately. However, they were included in the analyses for all adolescents and young adults.

To examine trends over time, the data sets from the Drug Affinity Studies and Alcohol Surveys from 2008 to 2023 were combined in a single data file and the 12-month prevalences with their 95 % confidence intervals were determined for 12- to 17-year-olds and 18- to 25-year-olds per survey year. Binary logistic regressions were used to estimate temporal trends. For this purpose, the independent variable ‘survey year’ was transformed to a value range starting with one. The survey year was then exponentiated and modelled stepwise as a polynomial of the first to sixth degree. This allows trend estimates that can be not only linear but also wave-shaped. Based on the Bayesian information criterion (BIC), the model with the best fit to the observed values was selected [18]. The logits of the regression equations were transformed into percentage values for each year and thus graphically represented as estimated trend curves of the prevalences [19].

Data management and analysis were performed using the IBM SPSS Statistics software package, version 30.0.0.0. Confidence intervals were calculated using the Complex Samples module.

### 2.4 Presentation of nationwide prevention measures – On-site, digital and analogue

The presentation focuses on all prevention measures that (1) the BIÖG has developed itself or promotes in Germany and that (2) directly or indirectly (i.e. via multipliers) target the prevention of cannabis use among young people in Germany. First, the subdivision and development basis of the programmes in schools and local communities is outlined, followed by a systematic presentation of characteristics such as access and settings, field of action, target groups, main methods and measures used for quality assurance and evaluation. This is followed by an overview of the BIÖG’s digital and analogue education and counselling programmes for cannabis prevention.

## 3. Results

### 3.1 Differences in cannabis use according to sociodemographic characteristics in 2023

A total of 7,001 adolescents and young adults aged 12 to 25 participated in the 2023 Drug Affinity Study, of whom 5 (0.1 %) did not provide any information on their cannabis use in the past twelve months. Of the remaining 6,996 respondents, 3,406 were adolescents aged 12 to 17 and 3,590 were young adults aged 18 to 25. Of the adolescents, 48.1 % were female, 65.0 % attended grammar school and 18.7 % had a migration background. Of the young adults, 41.6 % were female, 78.4 % had a university entrance qualification and 19.0 % had a migration background. In the unweighted sample, 12- to 17-year-olds were overrepresented. Since the weighting adjusts the age distribution of the total sample to the age distribution of all 12- to 25-year-olds in Germany, the weighted case numbers for young people are lower and those for young adults higher than the unweighted case numbers. The large proportion of respondents with higher education was also significantly corrected by weighting the data (Table 1 and Table 2).

Table 1 shows that 6.7 % of young people aged 12 to 17 had used cannabis in the twelve months prior to the survey (12-month prevalence). There was no significant difference between female and male adolescents in this regard. There were clear age differences within the group of adolescents. Of the 12- to 13-year-olds, 0.5 % had used cannabis in the last twelve months, compared with 2.6 % of 14- to 15-year-olds and 17.0 % of 16- to 17-year-olds. This significant increase with age was observed in both female and male adolescents. Cannabis use tended to be slightly more widespread among young people with higher education than among those with lower or medium education. However, this difference proved to be not significant within the two gender groups and in a binary logistic regression model with the covariates gender, age, education and migration background (not shown here, see [1]). Young people with and without a migration background did not differ in terms of the prevalence of cannabis use.

The results for young adults are shown in Table 2 and indicate that almost a quarter of 18- to 25-year-olds (23.5 %) had used cannabis in the last twelve months. There were significant gender differences in this age group. Young men had a significantly higher 12-month prevalence of cannabis use than young women (26.9 % vs. 19.4 %). Among young adults, there were no age differences or differences in terms of educational and migration status.

### 3.2 Changes in the prevalence of cannabis use by 2023

A total of 66,913 adolescents and young adults aged 12 to 25 participated in the eleven representative surveys conducted between 2008 and 2023. Of these, 102 (0.2 %) did not provide any information about their cannabis use in the last twelve months. The number of respondents with missing data ranged from 0 (2008) to 19 (2016) in the individual surveys. Table 3 shows the unweighted case numbers of female and male adolescents and young adults who provided valid data and could be included in the analyses for the individual surveys. Over time, the sample size was expanded, on the one hand to increase the statistical power of the studies and, on the other hand, due to the addition of mobile phone samples in the dual-frame approach from 2015 onwards.

Among female and male adolescents, the 12-month prevalence of cannabis use was lowest in 2011 during the period shown in Table 3. Cannabis use was most prevalent among female adolescents in 2021 and among male adolescents in 2019. Among young women and young men, cannabis use was lowest in 2008 and most prevalent in 2021.

**Figure 1:**
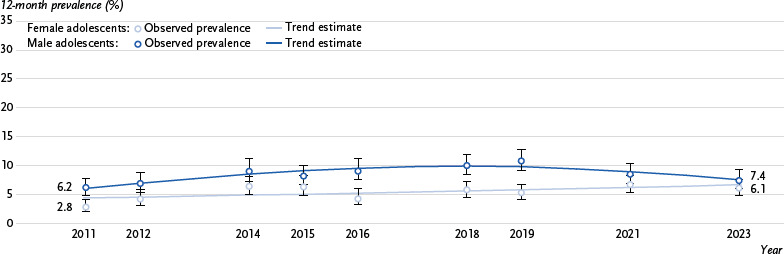
Trends in the 12-month prevalence of cannabis use among female and male adolescents from 2011 to 2023^*^. Source: Drug Affinity Studies 2011 – 2023, Alcohol Surveys 2010 – 2021 ^*^Trend estimate for male adolescents: Logit(cannabis use = 1) = – 2.928 + 0.183 (year – 2010) – 0.012 (year – 2010)^2^, Trend estimate for female adolescents: Logit(cannabis use = 1) = – 3.125 + 0.037 (year – 2010)

Figure 1 shows the observed prevalences with their 95 % confidence intervals and the trends estimated using logistic regression for female and male adolescents. The graph begins in 2011, the year in which the prevalence of use among adolescents was lowest (Table 3). Based on this minimum, estimates were made as to whether or not there were upward trends in the following years. For female adolescents, the best fit of the trends to the observed prevalences was achieved using a first-degree polynomial. According to this, their cannabis use rose slightly but significantly and steadily between 2011 and 2023. For male adolescents, a second-degree polynomial was estimated. This resulted in a reverse U-shaped trend, i.e. first an increase and then a decrease in the prevalence of cannabis use.

**Figure 2:**
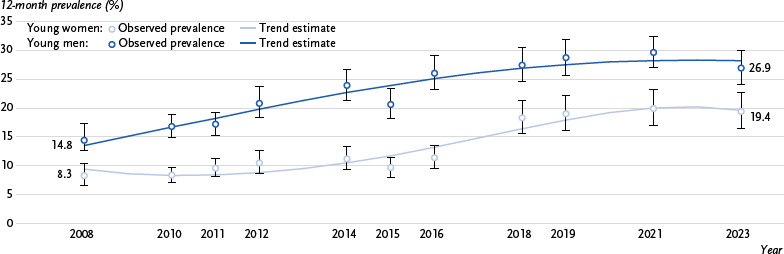
Trends in 12-month prevalence of cannabis use among young women and men from 2008 to 2023^*^. Source: Drug Affinity Studies 2008 – 2023, Alcohol Surveys 2010 – 2021 ^*^Trend estimate for young men: Logit(cannabis use = 1) = – 1.991 + 0.144 (year – 2007) – 0.005 (year – 2007)^2^, Trend estimate for young women: Logit (cannabis use = 1) = – 2.102 – 0.191 (year – 2007) + 0.036 (year – 2007)^2^ – 0.001 (year – 2007)^3^

For young men, too, the changes in the prevalence of cannabis use were best estimated using a second-degree polynomial (Figure 2). The graph shows an initial sharp increase, which flattens out over time. For young women, the best fit for the trends observed was a third-degree polynomial. From 2008 to the mid-2010s, cannabis use among young women changed little, then rose significantly until the end of the 2010s and changed only slightly thereafter.

### 3.3 Focus on cannabis prevention – Measures offered by the Federal Institute of Public Health

Behavioural prevention measures are aimed directly at individuals or groups. They aim to strengthen their knowledge, attitudes, skills and thus their behaviour in a way that promotes health. Contextual prevention measures focus on social living conditions and structures. These are to be changed in such a way that people can live healthier lives or are more likely to behave in a way that promotes health [20, 21].

Another distinction is how specific the prevention measure is and how at risk the target group is [20, 21]. Universal cannabis prevention is aimed at everyone, including those who have not yet used cannabis and do not (yet) intend to do so. Selective cannabis prevention is aimed specifically at young people who are at increased risk of starting to use cannabis. One example is young people with increased psychosocial stress, such as an unstable relationship with their parents or neglect [22, 23]. Indicated prevention is aimed at young people with initial signs of problematic behaviour, such as intensive cannabis use.

A further subdivision is into settings or fields of action such as family, school, community, media or legal framework conditions [21].

Finally, from the BIÖG’s point of view, it is important to consider whether the prevention measures are implemented by multipliers or whether the BIÖG addresses young people directly. The first case requires the qualification, training and support of multipliers as well as the willingness to cooperate and the commitment of third parties in the local settings. The second case requires directly reaching young people and publicising the measures. Then they can be easily accessed and used by young people themselves.

Current research findings are also taken into account in the development of appropriate prevention programmes. In this context, information and counselling services for young people and their adult caregivers on the subject of cannabis are continuously expanded, intensified and evaluated for their effectiveness.

The CanPräT research project (‘Inhalte, Settings und Zielgruppen der verhaltensbezogenen Cannabisprävention unter den Bedingungen der Teil-Legalisierung’), which systematically identified areas for improvement in existing cannabis-related programmes, projects and materials, came to the following conclusions based on North American evaluation studies [24, 25]:

1. ‘Awareness campaigns on the key legal provisions governing cannabis regulation should be carried out.
2. Cannabis education programmes for young people should be carried out, using interactive approaches to impart knowledge (including on harm minimisation) and minimise stigmatisation.
3. Primary prevention materials should be developed for non-users and secondary prevention materials for users.
4. Educational messages consistent with the principles of harm reduction should be communicated to young adults’ [24].

When developing and promoting services, the BIÖG also focuses on analysing data (see 3.1) – this shows which target groups need services. For example, among 18- to 25-year-olds, consumption is significantly higher among men, whereas among younger consumers there is no gender-specific pattern. There are also no significant differences based on educational or migration background. The combination of these results with scientific findings on reaching target groups (see CanPräT project) forms the basis for developing a strategy for action. Accordingly, one focus is on addiction prevention in schools, including vocational schools and universities, where young people can be easily reached.

#### Local services in schools and communities

In an ‘Expertise in Addiction Prevention 2020’ (‘Expertise zur Suchtprävention 2020’) commissioned by BIÖG [21], the prevention of illegal substances (at the time also cannabis prevention) was also examined by means of a systematic evaluation of reviews and meta-analyses on effective measures for addiction prevention. Among other things, it concludes that, in the family sphere, universal family-oriented prevention can delay the onset of cannabis use and reduce the frequency of cannabis use. In the school sphere, no specific method (such as the promotion of social skills or self-control strategies) is associated with stronger preventive effects among pupils in grades 8 to 12. However, drug prevention that follows the approach of promoting social skills (e.g. communication, self-confidence, problem-solving skills) or the model of social influence (e.g. actively rejecting consumption, resisting peer pressure, non-consuming role models) can have short-term preventive effects, and combining these approaches has preventive effects that last longer than a year.

The BIÖG takes these results into account in the planning and implementation of appropriate prevention measures. The focus is on measures in the school setting (interactive projects and teaching materials), as these particularly reach young people with no experience of consumption [26]. This enables young people to be made aware of the risks of cannabis use at an early stage and motivates them to consciously refrain from cannabis use. Appropriate evaluation of the projects forms the basis for quality-assured services. The evaluation stages range from process evaluations and impact evaluations to randomised controlled trials (RCTs). The process evaluation examines in particular the procedure, implementation and achievement of target groups. Impact evaluation serves to map the results and effects of the project [27]. In RCTs, intervention and control groups are examined in order to demonstrate the impact of a measure on this basis.

Table 4 provides an overview of prevention measures in schools, families and leisure time. The projects and their evaluation have been or are being funded by the BIÖG. The evaluation processes are at different stages of completion, and some are not yet finished.

#### Digital services, counselling and support

The digital measures focus on providing fact-based information to educate young people and strengthen their risk competence. This involves not only preparing information on legal regulations in a way that is appropriate for the target group, but also providing information on harm reduction (‘safer use’) for adults and support services for consumers to reduce their cannabis use or achieve abstinence. In addition, particularly for the purpose of quality assurance of the information provided, the BIÖG promotes the systematic collection and evaluation of ‘Cannabis – Risks and Myths’ (‘Cannabis – Risiken und Mythen’ – CaRM), the results of which are expected in 2025.

The BIÖG offers a total of three websites on the topic of cannabis prevention for different target groups, four social media channels and individual counselling services.

The landing page www.infos-cannabis.de was set up in October 2023 on the basis of the Cannabis Act (CanG) and provides an overview of nationwide cannabis prevention measures, information on the current legal situation, safer use recommendations for adult consumers and a referral function to the relevant prevention measures.

The website www.cannabispraevention.de takes a universal approach, providing information for young people aged 14 to 17 with no experience of cannabis use, as well as their adult caregivers such as parents, teachers and specialists. Young people are increasingly being addressed via the campaign’s various social media channels (Instagram, TikTok, YouTube) in order to reach them in ‘their’ environment with fact-based preventive messages.

The wide-reaching internet portal www.drugcom.de pursues a selective prevention approach and aims to prevent substance abuse and addiction and avert the negative consequences of substance use. The aim is to motivate young people between the ages of 15 and 25 who are interested in drugs to use psychoactive substances, including cannabis, in a low-risk and responsible manner, and to support them in reducing or ending their substance use. The portal is supplemented by the YouTube channel of the same name.

The portal www.drugcom.de offers both the digital selftest ‘Cannabis Check’, which provides users with quick, individual feedback on their own consumption behaviour, and the evidence-based, quality-assured and free behaviour change programme ‘Quit the Shit’. In addition, the portal offers email counselling and anonymous, free chat counselling.

Further counselling services are provided by the BIÖG in the form of telephone counselling. The addiction prevention information hotline offers answers to questions about addiction prevention, personal counselling for addiction problems with the aim of referring people to suitable local support services, and the names of local contact persons for assistance.

The ‘Addiction & Drugs Hotline’ (‘Sucht & Drogen Hotline’) pools the resources of regional drug emergency hotlines to create an effective network of mutually supportive addiction support services. The hotline offers personal advice, support and information from experienced addiction support professionals for both people with addiction disorders and their relatives.

The BIÖG’s prevention measures are supplemented by printed materials that are made available free of charge to various target groups. In the area of cannabis prevention, places a strong focus on the provision and further development of teaching materials for use in schools.

## 4. Discussion

The BIÖG study results show a 12-month prevalence of cannabis use in 2023 of 6.7 % among adolescents aged between 12 and 17 and 23.5 % among young adults aged between 18 and 25. When comparing with other studies from Germany and Europe, different survey methods, survey periods and age groups represented must be taken into account. Looking at the group of adolescents, representative data for Germany is available from the 2020 study Improving Mental Health and Reducing Addiction in Childhood and Adolescence through Mindfulness: Mechanisms, Prevention and Treatment (IMAC-Mind), in which adolescents aged between 12 and 18 were surveyed. The survey was conducted by telephone and reported a similarly high 12-month prevalence of cannabis use of 6.6 % [16]. Representative data for Europe is available from the European School Survey Project on Alcohol and Other Drugs (ESPAD) from 2024. 15- to 16-year-olds from 37 European countries completed a questionnaire on their substance use online or on paper. On average, the 12-month prevalence of cannabis use among the participating ESPAD countries was 9.0 %, with Germany in the upper mid-range with data from Bavaria (12-month prevalence of 14.2 %) [35].

For adults aged 18 to 24 in Germany, the Epidemiological Survey on Substance Abuse (ESA), which collected data in writing, online or by telephone [36], found a similar 12-month prevalence of 23.9 % for 2021 from [37], which is consistent with the results of the Drug Affinity Study for young adults. The DEBRA study (German Study on Tobacco Use) used data from computer-assisted personal interviews in households in 2022/2023 to arrive at a lower 12-month prevalence of cannabis use of 11.4 % for the population in Germany aged 14 to 24 [38], while the 2023 Drug Affinity Study showed a 12-month prevalence of 18.8 % for the age group 14 to 24 years.

Female and male adolescents did not differ statistically significantly in our results in 2023, which is also consistent with the results of the IMAC-Mind study for 2020 [16]. Prior to this time, gender differences were still observed among adolescents. In the years up to and including 2019, significantly more boys than girls used cannabis. This was evident not only in the BIÖG data, but also, for example, in the HBSC (Health Behaviour in School-Aged Children) study from 2017/2018 for 15-year-old schoolchildren [39]. The fact that girls and boys have converged in terms of cannabis use in recent years was also described in the ESPAD study for Europe [40]. Furthermore, the available data showed a significant increase in cannabis use among young people as they got older. This can be attributed to social opportunities and typical developmental tasks during adolescence on the path to adulthood [2, 3]. The findings also highlight the importance of implementing universal cannabis prevention measures before the age of 16. Other sociodemographic characteristics examined here, such as educational attainment and migration background, were not significantly associated with cannabis use among adolescents. This is also largely consistent with the results of other German studies [16]. For example, the HBSC study mentioned above found that only girls with a unilateral migration background had an increased risk of cannabis use [39].

In the group of young adults, on the other hand, there were significant gender differences in the 12-month prevalence of cannabis use, with significantly more young men than young women using cannabis (26.9 % vs. 19.4 %). These gender differences were found in many studies both in Germany [14, 36, 38] and internationally [41, 42]. No differences were found among young adults in terms of age, educational status or migration status.

The results presented here are based on data collected before the Consumer Cannabis Act came into force. Until then, there had been little change in the trend among young people over the fifteen years from 2008 to 2023. Among male adolescents, cannabis use actually declined between 2019 and 2023. A similar trend was observed for 15- to 16-year-old school pupils in Europe in the ESPAD data (covering the period from 2007 to 2024) [35].

Among young adults, on the other hand, cannabis use rose significantly, reaching peak levels in 2021 for both young women and young men. Between 2021 and 2023, cannabis use among young adults did not increase further, but remained at a high level. For Germany, data from the ESA for the age group 18 to 59 years also show a peak in 12-month prevalence among women and men in 2021 [43], and the trend among 18- to 24-year-olds in the ESA is very similar to that among 18- to 25-year-olds in the Drug Affinity Studies [37, 44]. For Europe, increases in 12-month prevalence for people aged 15 to 64 were identified in the decade between 2010 and 2019, with the largest increases observed in the older age groups [45].

The results underscore the importance of providing understandable and easily accessible information and prevention measures. In particular, educating people about the health risks and other negative consequences associated with cannabis use is a key component of this effort. For example, the results available for 2023 show that more than half of adolescents and about one-third of young adults rated their level of information about the effects of cannabis use as ‘not very good’ or ‘not good at all’ [1]. Low-threshold services and diverse access channels to reach target groups in different living environments are therefore of great importance. In this context, possible consumption trends, mental comorbidities and possible mixed consumption will continue to be taken into account.

The BIÖG focuses on the data- and evidence-based development of prevention measures (see 3.1). Based on scientific findings and relevant study results, such as the Drug Affinity Study, new and further development needs are analysed and optimisation potential and possible trends are identified. Table 4 shows that young people aged 14 and above in particular are reached by a wide range of universal prevention measures. This is relevant in light of the fact that first-time cannabis use often occurs during adolescence, as young people should be made aware of the risks of consumption as early as possible and motivated to use cannabis responsibly. Schools are particularly well suited for this purpose, as they enable multipliers such as parents and educational professionals to implement prevention measures. This requires low-threshold and practical training measures – one example is the online course ‘Cannabis Prevention: Knowledge, Understanding, Action’ (‘Cannabisprävention: Wissen, verstehen, handeln’), which is available digitally and free of charge. With the strengthening of cannabis prevention measures following the entry into force of the Cannabis Act in 2024, nationwide cannabis prevention measures have been developed, expanded, piloted, evaluated and consolidated in cooperation between the federal and state governments. In addition to the BIÖG’s offerings, there are a wide range of quality-assured prevention measures, and the focus should continue to be on networking, jointly establishing and providing suitable nationwide coverage of these measures.

At the same time, measures to strengthen individual social and emotional skills are relevant at all ages. As a rule, these so-called life skills measures start in childhood and usually take a substance-independent approach, meaning they do not focus on substances such as cannabis. Nevertheless, approaches to promoting life skills can be found in the projects described in 3.3. Their aim is to strengthen young people in the areas of self-awareness, empathy, critical thinking, problem-solving skills and stress management, thereby enabling them to actively reject substance use. In the future, measures to strengthen life skills in childhood and adolescence will need to be further focused and intensified – for example, people who wish to have children and pregnant women should also be taken into account, as the foundations of life skills are already laid prenatally.

## Limitations

The present results are based on self-reported information from the respondents about cannabis use, which was still illegal at the time. Recording illegal behaviour in research is difficult and carries the risk that respondents will give socially desirable answers and tend to conceal illegal behaviour. It cannot therefore be ruled out that the prevalence of cannabis use is underestimated in the available data. For example, a study of young adults in Sweden found a significantly higher 12-month prevalence of cannabis use when respondents were only asked indirectly about their drug use [46]. Studies that compared self-reported drug use with laboratory values (e. g. hair or urine analyses) from respondents came to very different conclusions, with minor but also major discrepancies in the values regarding cannabis use (for an overview, see [47]). Furthermore, the trends reported here may have been influenced by the COVID-19 pandemic. However, study results for the adult population in Germany suggest that consumers largely did not change their consumption behaviour during the pandemic [43].

## Conclusions

The present findings shed light on cannabis use trends among 12- to 25-year-olds before the Consumer Cannabis Act came into force. While there were only minor changes in cannabis use among adolescents, significantly more young women and men used cannabis in 2023 than in 2008. The increases among young adults occurred during periods when cannabis was illegal and have recently slowed down. It remains to be seen whether and how the consumption behaviour of adolescents and young adults will change in the wake of legalisation for adults after 2024. Future representative surveys – such as the present one – can provide important insights for the evaluation of the Consumer Cannabis Act.

## Figures and Tables

**Table 1:** 12-month prevalence of cannabis use among adolescents aged 12 to 17 years by gender, age, education and migration background in 2023. Source: Drug Affinity Study 2023

	Young people overall				Female adolescents				Male adolescents			
	Number of cases		Prevalence		Number of cases		Prevalence		Number of cases		Prevalence	
	Unweighted^*^	Weighted	%	(95 % CI)	Unweighted	Weighted	%	(95 % CI)	Unweighted	Weighted	%	(95 % CI)
	3,406	2,768	6.7	(5.7 – 7.9)	1,638	1,337	6.1	(4.9 – 7.5)	1,761	1,422	7.4	(5.8 – 9.3)
**Age group**												
12 and 13 years	1,092	924	0.5	(0.2 – 1.4)	515	447	1.1	(0.4 – 2.8)	576	475	0.0	(0.0 – 0.0)
14 and 15 years	1,143	921	2.6	(1.7 – 3.9)	556	445	2.5	(1.4 – 4.3)	585	472	2.8	(1.5 – 4.9)
16 and 17 years	1,171	923	17.0	(14.3 – 20.1)	567	445	14.6	(11.5 – 18.4)	600	474	19.5	(15.2 – 24.5)
**Education**												
Low/medium	1,192	1,681	5.4	(4.0 – 7.2)	497	742	4.5	(3.0 – 6.8)	691	933	6.1	(4.1 – 9.0)
High	2,214	1,087	8.8	(7.5 – 10.3)	1,141	595	7.9	(6.2 – 10.0)	1,070	489	9.9	(8.0 – 12.1)
**Migration background**												
No	2,769	2,246	6.6	(5.5 – 7.9)	1,364	1,107	6.1	(4.7 – 7.7)	1,399	1,131	7.2	(5.5 – 9.3)
Yes	637	521	7.3	(5.0 – 10.6)	274	229	6.1	(3.5 – 10.3)	362	291	8.3	(5.0 – 13.5)

^*^Including adolescents with the gender category ‘diverse’ (n = 7)

95 % CI = 95 % confidence interval

**Table 2:** 12-month prevalence of cannabis use among young adults aged 18 to 25 by gender, age, education and migration background in 2023. Source: Drug Affinity Study 2023

	Young adults overall				Young women				Young men			
	Number of cases		Prevalence		Number of cases		Prevalence		Number of cases		Prevalence	
	Unweighted^*^	Weighted	%	(95 % CI)	Unweighted	Weighted	%	(95 % CI)	Unweighted	Weighted	%	(95 % CI)
	3,590^*^	4,226	23.5	(21.4 – 25.6)	1,493	2,009	19.4	(16.5 – 22.6)	2,081	2,202	26.9	(24.1 – 29.9)
**Age group**												
18 and 19 years	1,174	943	24.4	(21.3 – 27.7)	544	452	20.8	(16.8 – 25.6)	624	488	27.3	(22.8 – 32.2)
20 and 21 years	930	1,026	22.2	(18.7 – 26.2)	393	488	15.0	(11.2 – 19.9)	531	533	28.7	(23.1 – 35.0)
22 and 23 years	842	1,097	23.0	(18.8 – 27.6)	332	518	21.1	(15.1 – 28.7)	507	574	24.4	(19.1 – 30.5)
24 and 25 years	644	1,161	24.3	(19.8 – 29.4)	224	551	20.4	(14.1 – 28.6)	419	607	27.4	(21.5 – 34.2)
**Education**												
Low/medium	775	1,715	22.4	(18.5 – 26.9)	221	605	18.3	(11.9 – 27.0)	553	1,109	24.6	(19.9 – 30.0)
High	2,815	2,511	24.2	(22.2 – 26.3)	1,272	1,404	19.9	(17.2 – 22.9)	1,528	1,092	29.2	(26.5 – 32.0)
**Migration background**												
No	2,908	3,336	23.5	(21.2 – 25.9)	1,226	1,616	20.1	(16.8 – 23.8)	1,670	1,708	26.3	(23.2 – 29.7)
Yes	682	890	23.5	(19.3 – 28.2)	267	393	16.6	(11.8 – 22.9)	411	494	28.8	(23.0 – 35.6)

^*^Including young adults with the gender category ‘diverse’ (n = 16)

95 % CI = 95 % confidence interval

**Table 3:** 12-month prevalence of cannabis use and number of cases from studies from 2008 to 2023*. Source: Drug Affinity Studies 2008–2023, Alcohol Surveys 2010–2021

		2008	2010	2011	2012	2014	2015	2016	2018	2019	2021	2023
**Adolescents**												
Female	Prevalence (%)	4.4	3.2	2.8	4.2	6.4	6.3	4.5	5.8	5.3	6.7	6.1
	(95% CI)	(3.0–6.4)	(2.2–4.4)	(2.0–4.0)	(3.1–5.8)	(5.0–8.2)	(4.8–8.2)	(3.4–5.9)	(4.6–7.2)	(4.2–6.6)	(5.4–8.3)	(4.9–7.5)
	Number of cases	608	1,280	997	1,029	970	1,583	1,552	1,536	1,651	1,538	1,638
Male	Prevalence (%)	8.7	6.7	6.2	6.9	9.0	8.1	9.3	10.0	10.8	8.5	7.4
	(95% CI)	(6.7–11.2)	(5.3–8.4)	(4.9–7.8)	(5.4–8.8)	(7.3–11.1)	(6.6–10.0)	(7.7–11.2)	(8.4–11.9)	(9.2–12.7)	(6.9–10.3)	(5.8–9.3)
	Number of cases	612	1,297	1,035	996	1,048	1,710	1,638	1,646	1,780	1,565	1,761
**Young adults**												
Female	Prevalence (%)	8.3	8.4	9.6	10.5	11.2	9.7	11.4	18.3	19.0	19.9	19.4
	(95% CI)	(6.6–10.3)	(7.1–9.8)	(8.2–11.2)	(8.7–12.7)	(9.4–13.3)	(8.1–11.5)	(9.5–13.5)	(15.6–21.2)	(16.1–22.2)	(17.0–23.1)	(16.5–22.6)
	Number of cases	903	2,216	1,480	1,460	1,361	1,718	1,658	1,683	1,504	1,580	1,493
Male	Prevalence (%)	14.8	16.8	17.2	20.8	23.9	20.6	26.0	27.4	28.7	29.6	26.9
	(95% CI)	(12.6–17.3)	(15.0–18.8)	(15.3–19.2)	(18.3–23.6)	(21.3–26.7)	(18.1–23.4)	(23.2–29.1)	(24.6–30.4)	(25.6–31.9)	(26.9–32.4)	(24.1–29.9)
	Number of cases	878	2,198	1,485	1,505	1,507	1,981	2,136	2,127	2,055	2,306	2,081

*The figures show the unweighted number of cases and weighted prevalences in percent with their 95 *%* confidence intervals (95 % CI).

**Table 4:** Cannabis prevention measures funded by the Federal Institute of Public Health (as of July 2025). Source: Federal Institute of Public Health

Prevention measures	Multipliers	Specificity	Field of action	Target group	Main method	Evaluation	On the internet (in German)
Der Grüne Koffer – Methodenset Cannabisprävention	Teachers, school social workers	Universal	School	Pupils from Year 8 onwards	Interactive workshop	RCT; Isensee et al.[28]	https://www.starkstattbreit.nrw.de/Gruener-Koffer
(Virtuelle) Elternabende zur suchtpräventiven Information zum Thema Cannabis (ESIC)	Teachers	Universal	School/family	Parents of pupils from Year 8 onwards	Parents‘ evening	Process evaluation; not yet completed	https://www.cannabiselt.ernabend.de/
Cannabis – Quo Vadis?	Teachers, specialists in (addiction) prevention	Universal	School	Pupils from Year 8 onwards	Interactive workshop	RCT; Suchert et al. [29]	https://www.villa-schoepflin.de/cannabis-quo-vadis.html
Meine Zeit ohne – Die Challenge (MZo)	Teachers	Universal/selective	(Vocational) school	Pupils from Year 9 onwards	Joint challenge with consumption reduction/renunciation	RCT; Pietsch et al. [30]	https://www.meine-zeit-ohne.de/
Cannabis Kompakt	Teachers	Universal	School	Pupils from Year 8 onwards	Three teaching units	RCT; not yet completed	https://www.cannabis-kompakt.de/
InstaVention	Teachers, specialists in (addiction) prevention	Universal	School	15- to 21-year-olds	Instagram account and accompanying booklet for interactive knowledge transfer	Effectiveness evaluation; not published	https://www.suchtgeschichte.nrw.de/Kampagne/InstaVention
Escape Cannabis	Teachers	Universal	School	14- to 18-year-olds	Digital game for knowledge transfer	Process evaluation; not yet completed	https://escape-cannabis.de/
REBOUND 2.0	Teachers	Universal	(Vocational) school, university	18- to 25-year-olds	16-part course on promoting skills and resilience	Impact evaluation; evaluation of REBOUND 2.0 not yet completed, results of a pilot study on REBOUND: Jungaberle et al. [31]	https://rebound.schule/ and https://finder-akademie.de/projekte/rebound-2-0/
IPSY 8 CAN	Teachers	Universal	School	Pupils from Year 8 onwards	Life skills programme with knowledge transfer on cannabis	Process evaluation; evaluation of ‘IPSY 8 CAN’ not yet completed, evaluation of ‘IPSY’: quasiexperimental control group design; Weichold et al. [32]	https://www.ipsy.uni-jena.de/
Cannabisprävention – Wissen, verstehen, handeln	Teachers, specialists in (addiction) prevention, parents	Further training	School	Teenagers, young adults, consumers	Online course	Process evaluation; not published	https://www.zpg-bayern.de/online-kurs-cannabispraevention.html
Frühintervention bei Drogenkonsum in der Adoleszenz (FriDA)	Addiction counsellors	Indicated	Family	Adolescents and young adults, including their families	Family therapy and systemic support for adolescents with substance use problems	Process evaluation; Gantner et al. [33]	https://www.frida-beratung.de/
MOVE Cannabis	Specialists	Indicated	School/leisure/Youth welfare	Young people who use cannabis	Training for professionals in motivational interviewing when dealing with young people who are unwilling to change	Evaluation of ‘MOVE Cannabis’ is being coordinated; Process evaluation of ‘MOVE’ [34]	https://www.move-seminare.de/Jugendliche/MOVE-Cannabis

RCT = Randomised Controlled Trial

## Footnotes

^1^ Article 1 of the Act on the Controlled Use of Cannabis and on the Amendment of Other Regulations (Cannabis Act – CanG) of 27 March 2024, Federal Law Gazette 2024 I No. 109, last amended by Article 1 of the Act of 20 June 2024 (Federal Law Gazette 2024 I No. 207).

^2^ The Federal Centre for Health Education (BZgA) was renamed to the Federal Institute of Public Health (BIÖG) on 13 February 2025.
